# Dataset of mechanical properties of coarse aggregates stabilized with traditional and nontraditional additives: Stiffness, deformation, resistance to freezing and stripping

**DOI:** 10.1016/j.dib.2022.108781

**Published:** 2022-11-24

**Authors:** Diego Maria Barbieri, Baowen Lou, Robert Jason Dyke, Hao Chen, Umesh Chandra Sahoo, Jeb S Tingle, Inge Hoff

**Affiliations:** a Norwegian University of Science and Technology, Department of Civil and Environmental Engineering. Høgskoleringen 7A, Trondheim, 7491, Trøndelag, Norway; b Chang'an University, School of Highway. Nan Er Huan Road (Mid-section), Xi'an, 710064, Shaanxi, China; c Oslo Metropolitan University, Department of Civil Engineering and Energy Technology. Pilestredet 35, Oslo 0166 Norway; d Indian Institute of Technology Bhubaneswar, School of Infrastructure. Arugul, Jatni, 752050, Odisha, India; e U.S. Army Engineer Research and Development Center, Geotechnical and Structures Laboratory. 3909 Halls Ferry road, Vicksburg, 39180, Mississippi, United States

**Keywords:** Road stabilization, Traditional stabilizers, Nontraditional stabilizers, Unbound granular materials, Pavement geotechnics, Repeated load triaxial test, Freeze-thaw cycles, Rolling bottle test

## Abstract

The dataset derives from a thorough laboratory characterization of all existing stabilization technologies suitable for coarse-graded aggregates. They include two traditional binders (based on cement and bitumen) and eleven nontraditional binders (based on brine salt, clay, organic non-petroleum, organic petroleum and synthetic polymer). The dataset derives from four laboratory test operations: repeated load triaxial test performed both before and after exposing the investigated samples to ten freeze-thaw cycles, weight measurement of Marshall specimens during ten freeze-thaw cycles and a modified version of rolling bottle test. Repeated load triaxial tests assess the resilient modulus and the resistance to permanent deformation of both unstabilized and stabilized specimens. The mass loss of Marshall specimens expresses the susceptibility of each additive to lose its binding property when exposed to freezing action. The modified version of the rolling bottle test characterizes the propensity to stripping for each additive coating the aggregates subjected to mechanical stirring action. Given the surging necessity to improve the construction and maintenance operations for road pavements worldwide, this dataset containing information about several stabilization technologies can be very useful for transport agencies, contractors, industry and university researchers as well as companies manufacturing and supplying stabilization technologies.

## Graphical abstract

**Figure:**
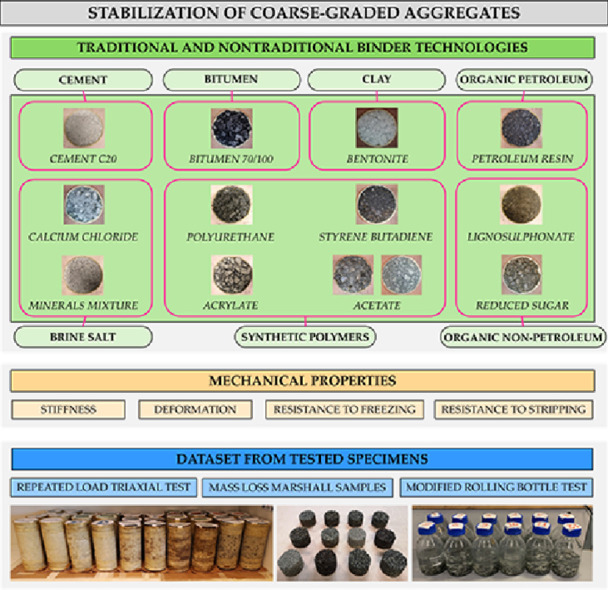

**Specification Table**

**Table:** 

Subject	Civil and Structural Engineering
Specific subject area	Road stabilization, Traditional stabilizers, Nontraditional stabilizers, Unbound granular materials, Pavement geotechnics, Freeze-thaw cycles, Repeated load triaxial test, Rolling bottle test
Type of data	Table Image
How data were acquired	The data were collected performing the following laboratory tests: Repeated Load Triaxial Test (RLTT) both before and after the action of 10 Freeze-Thaw (FT) cycles, weight measurement of Marshall specimens during 10 FT cycles and a modified version of Rolling Bottle Test (RBT) for 14 different time intervals, namely 1 h, 2 h, 3 h, 4 h, 5 h, 6 h, 7h, 8 h, 10 h, 12 h, 14h, 16 h, 20 h and 24 h. The total number of samples created and tested was: 28 RLTT samples (2 replicates, particle size between 0 mm and 32 mm), 39 Marshall samples (3 replicates, particle size between 4 mm and 8 mm) and 588 RBT samples (3 replicates, particle size between 8 mm and 11.2 mm).
Data format	Raw
Description of data collection	The research comprised all existing types of traditional and nontraditional stabilizers for coarse aggregates. Creation and testing of RLTT, Marshall and RBT samples were based on the codes “EN 13286-7 Cyclic load triaxial test for unbound mixtures”, “EN 12697-30 Specimen preparation by impact compactor” and “EN 12697-11 Determination of the affinity between aggregate and bitumen”, respectively.
Data source location	The testing campaign was performed at the Department of Civil and Environmental Engineering, Norwegian University of Science and Technology (NTNU), Høgskoleringen 7A, Trondheim 7491, Norway. Aggregates were collected from Vassfjell, Heimdal, Norway. All the stabilization technologies were kindly supplied by industrial producers (refer to Acknowledgments section).
Data accessibility	Dataset is uploaded on Mendeley Data Repository name: Mechanical properties of coarse aggregates stabilized with traditional and nontraditional additives: stiffness, deformation, resistance to freezing and stripping Data identification number: DOI: 10.17632/xvb2dtjdch.1 Direct URL to data: https://data.mendeley.com/datasets/xvb2dtjdch
Related research articles	D. M. Barbieri, B. Lou, R. J. Dyke, H. Chen, F. Wang, B. Dongmo-Engeland, J. S. Tingle and I. Hoff. Stabilization of coarse aggregates with traditional and nontraditional additives. Journal of Materials in Civil Engineering, 2022. https://doi.org/10.1061/(ASCE)MT.1943-5533.0004406 D. M. Barbieri, B. Lou, R. J. Dyke, X. Wang, H. Chen, B. Shu, U. Gazder, S. Horpibulsuk, J. S. Tingle and I. Hoff. Design and sustainability analyses of road base layers stabilized with traditional and nontraditional additives. Journal of Cleaner Production, 2022. https://doi.org/10.1016/j.jclepro.2022.133752

## Value of the Data

• Connected with the surging necessity to improve the construction and maintenance operations for road pavements worldwide, this dataset derives from a thorough laboratory characterization and comparison of all the existing binder technologies suitable for stabilizing coarse-graded aggregates.
• Considering the global relevance of road infrastructures, the dataset derived from an independent investigation of several stabilization technologies can become a very useful resource for transport agencies, contractors, industry and university researchers as well as companies manufacturing and supplying stabilization technologies.
• The data can be used to evaluate and compare the mechanical behavior associated to all the types of existing binder technologies that can stabilize coarse-graded aggregates. Furthermore, the data can be interpreted according to various regression models and their analysis can indicate the directions for possible further laboratory or field tests.
• As unpaved low-volume roads form most of the road infrastructures worldwide and are often in very poor condition due to lack of maintenance operations, the dataset formed in this single independent laboratory testing campaign represents a valuable resource for objective comparison across several stabilization technologies.

## 1 Objective

This work reports on the dataset “Mechanical properties of coarse aggregates stabilized with traditional and nontraditional additives: stiffness, deformation, resistance to freezing and stripping” (https://data.mendeley.com/datasets/xvb2dtjdch) [1] obtained by means of laboratory tests performed at the Department of Civil and Environmental Engineering (Norwegian University of Science and Technology, Trondheim, Norway) in 2020 and 2021. The major reason for performing such testing campaign has been to investigate and compare the stabilization potential of traditional and nontraditional binders that are used in road pavement engineering. Very few previous investigations have comprehensively compared the mechanical properties of both traditional and nontraditional technologies in a single independent study. The dataset has a size of 3 772 MB and revolves around the binder application to coarse-graded aggregates as thoroughly discussed in the two corresponding research articles [2,3]. The objective of this data article is three-fold: to describe how the data have been obtained in the laboratory, to explain the taxonomy adopted to store the dataset on the public repository Mendeley Data and to highlight how stakeholders (e.g., transport agencies, contractors, researchers, manufacturers of the stabilizers) can benefit from it.

## 2 Data Description

The dataset derives from a thorough investigation assessing the mechanical properties of all the existing binder technologies suitable for stabilizing coarse-graded aggregates [2,3]. The data were collected by performing four laboratory test operations: Repeated Load Triaxial Test (RLTT) both before and after the action of 10 Freeze-Thaw (FT) cycles, weight measurement of Marshall specimens during 10 FT cycles and a modified version of Rolling Bottle Test (RBT). The content of the dataset includes both raw data and pictures for all the tested specimen (https://data.mendeley.com/datasets/xvb2dtjdch) [1]. As reported in Table 1, thirteen stabilization technologies are investigated and represent both traditional additives (cement and bitumen) and nontraditional additives (brine salt, clay, organic non-petroleum, organic petroleum and synthetic polymer) [4], [5], [6], [7].

**Table 1:** Overview of the tested stabilization technologies and name abbreviations.

Numbering	Name of stabilization technology	Abbreviation
1	unbound granular material (untreated)	UGM
2	cement	CEM
3	bitumen	BIT
4	brine salt type A, calcium chloride	SAL-A
5	brine salt type B, minerals mixture	SAL-B
6	bentonite	BEN
7	lignosulphonate	LIG
8	reduced sugar	SUG
9	petroleum resin	RES
10	polyurethane	POL
11	acrylate	ACR
12	styrene butadiene	STB
13	acetate, type A	ACE-A
14	acetate, type B	ACE-B

### 2.1 Data from repeated load triaxial tests

The data derived from RLTTs are reported in the folder “Data from RLTT before 10 FT cycles” and “Data from RLTT after 10 FT cycles”. 14 subfolders contain the data for each stabilization treatment listed in Table 1, two replicate specimens (denoted as “01” and “02”) were tested for each additive binder. RLTT samples created using UGM materials were only tested before the exposure to 10 FT. For each specimen, three pieces of information are reported according to the nomenclature reported in Table 2.

**Table 2:** Content of each subfolder referring to the samples tested with Repeated Load Triaxial Tests.

Specimen 01	
File type	File name
Spreadsheet with raw data (.xlsx)	“Spec. #*abbreviation*# 01”
Sample picture (.jpg)	“Spec. #*abbreviation*# 01 a”
Sample picture (.jpg)	“Spec. #*abbreviation*# 01 b”

Specimen 02	

File type	File name

Spreadsheet with raw data (.xlsx)	“Spec. #*abbreviation*# 02”
Sample picture (.jpg)	“Spec. #*abbreviation*# 02 a”
Sample picture (.jpg)	“Spec. #*abbreviation*# 02 b”

The content of all the spreadsheets is structured according to the same logic as described elsewhere [8]. Each spreadsheet contains five sheets (“Sequence 1”, “Sequence 2”, “Sequence 3”, “Sequence 4”, “Sequence 5”) corresponding to as many RLTT loading sequences. Each loading sequence is made of six steps and their number is reported in column A. The time *t* since the sequence started, temperature *T* (namely room temperature), deviatoric pulse number and frequency *f* (fixed to 10 Hz) are listed in column B, C, D and E, respectively. In the RLTT device the specimen is subjected to a triaxial stress state achieved by means of a hydraulic piston acting vertically and pressurized water acting in all the directions. The deviatoric stress *σ_d_* exerted by the hydraulic piston is made of two components, a dynamic part (*σ_d,dyn_*) and a static part (*σ_d,st_*); the values of the former one are reported in column F and the values of the latter one (always approximately equal to 5 kPa) are shown in column G. In a similar way for the triaxial stress *σ_t_*, the dynamic part (*σ_t,dyn_*, always approximately equal to 0 kPa) and the static part (*σ_t,st_*) are specified in columns H and I, respectively. Six Linear Variable Displacement Transformers (LVDTs) measure the deformations of the sample. Three LVDTs assess the vertical deformations classified as elastic components (*ε_a,el,01_, ε_a,el,02_, ε_a,el,03_*) and plastic components (*ε_a,pl,01_, ε_a,pl,02_, ε_a,pl,03_*); their values are reported in columns J, L, N and in columns K, M, O, respectively. Similarly, three LVDTs probe the radial deformations referred to as elastic components (*ε_r,el,01_, ε_r,el,02_, ε_r,el,03_*) and plastic components (*ε_r,pl,01_, ε_r,pl,02_, ε_r,pl,03_*); they are reported in columns P, R, T and in columns Q, S, U, respectively.

**Fig. 1:**
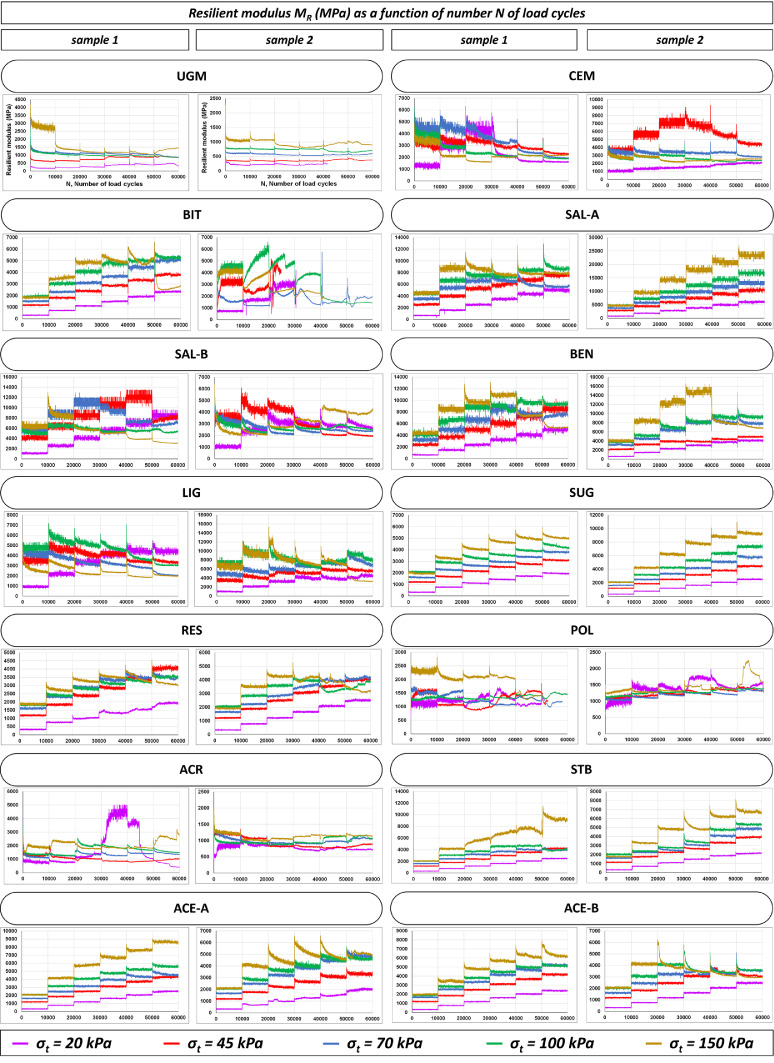
Experimental values of resilient modulus *M_R_* for all RLTT samples tested before the exposure to FT actions.

**Fig. 2:**
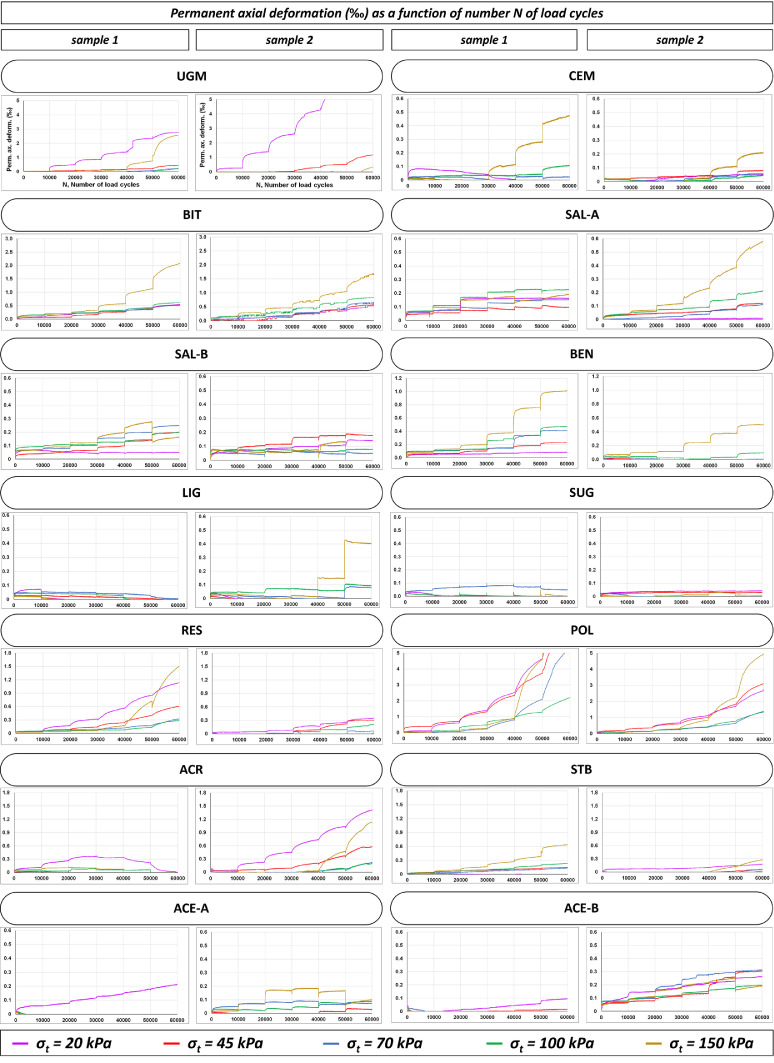
Experimental values of axial plastic deformation for all RLTT samples tested before the exposure to FT actions.

The two main mechanical properties assessed by RLTTs are the resilient modulus *M_R_* (definition reported in Section 2) and the resistance against permanent deformation. For an instance, the *M_R_* and the development of axial plastic deformation for all the RLTT specimens tested before the exposure to 10 FT is displayed in Figs. 1 and 2, respectively, according to the number of load cycles *N*; each colour corresponds to one of the five loading sequences (*σ_t_* = 20 kPa, 45 kPa, 70 kPa, 100 kPa, 150 kPa).

The raw data contained in the dataset can be further processed according to several regression expressions to analyse the experimental findings [9], [10], [11], [12], [13], [14]. In example, *M_R_* can be modelled according to the formulations proposed by Hicks & Monismith [15], Uzan [16] or Uzan & Witczak [17]. The development of the permanent deformation can be modelled according to the expressions developed by Barksdale [18], Sweere [19], Hyde [20] or Shenton [21].

### 2.2 Data from mass loss of Marshall samples

The data describing the mass loss of Marshall samples during the exposure to 10 FT cycles are contained in the folder “Data from Marshall samples during 10 FT cycles” which includes two files, namely “Weight of Marshall specimens.xlsx” and the folder “Marshall sample pictures”. The spreadsheet file “Weight of Marshall specimens.xlsx” contains the dried weight of all the samples after each FT cycle (1 FT, 2 FT, 3 FT, 4 FT, 5 FT, 6 FT, 7 FT, 8 FT, 9 FT and 10 FT, reported in columns B, Q, AG, AV). Three replicate specimens are tested for each binder additive and are named “1”, “2” and “3”. The abbreviations of the used stabilization technologies are reported in line 2. Based on these data, it is possible to assess the mass loss *ML_MRS_* (parameter defined in Section 2) as listed in Table 3. The samples stabilized with SAL-A and BEN were not tested as they were significantly damaged during the extraction from the mould and totally collapsed when exposed to water. The specimens containing BIT collapsed after a few FT repetitions due to the absence of fine aggregates and filler. The samples mixed with LIG and SUG gradually collapsed during the FT cycles.

**Table 3:** Experimental data of ML_MRS_ for each different binder technology.

	ML_MRS_ (%) for different FT cycles									
	FT1	FT2	FT3	FT4	FT5	FT6	FT7	FT8	FT9	FT10
CEM	0.07	0.10	0.10	0.16	0.19	0.31	0.33	0.35	0.38	0.39
BIT	14.75	23.38	36.96	41.76	*samples collapse*					
SAL-A	*samples collapse*									
SAL-B	0.24	0.28	0.30	0.34	0.35	0.42	0.43	0.45	0.51	0.54
BEN	*samples collapse*									
LIG	7.78	30.18	63.45	69.78	76.25	*samples collapse*				
SUG	19.74	36.66	65.54	68.64	74.19	*samples collapse*				
RES	4.29	5.65	7.14	10.62	14.19	15.69	16.84	17.98	19.12	21.61
POL	0.08	0.09	0.10	0.11	0.11	0.13	0.13	0.14	0.14	0.14
ACR	0.07	0.17	0.22	0.28	0.31	0.33	0.37	0.41	0.43	0.44
STB	0.03	0.08	0.09	0.11	0.11	0.14	0.14	0.15	0.16	0.16
ACE-A	0.04	0.05	0.06	0.06	0.06	0.08	0.08	0.09	0.09	0.09
ACE-B	0.03	0.07	0.08	0.08	0.08	0.10	0.11	0.11	0.12	0.12

The folder “Marshall sample pictures” contains 13 subfolders that display the images in .jpg format of each dried specimen for every FT cycle. Each subfolder contains up to 33 images according to the designation presented in Table 4. Thus, it is also possible to visually assess the integrity of each sample for different amounts of FT repetitions.

**Table 4:** Designation of Marshall samples and corresponding images for each FT cycle.

FT cycle	Specimen name			Name of corresponding image		
initial	1	2	3	“FT cycle 00, sample 1.jpg”	“FT cycle 00, sample 2.jpg”	“FT cycle 00, sample 3.jpg”
1	1	2	3	“FT cycle 01, sample 1.jpg”	“FT cycle 01, sample 2.jpg”	“FT cycle 01, sample 3.jpg”
2	1	2	3	“FT cycle 02, sample 1.jpg”	“FT cycle 02, sample 2.jpg”	“FT cycle 02, sample 3.jpg”
3	1	2	3	“FT cycle 03, sample 1.jpg”	“FT cycle 03, sample 2.jpg”	“FT cycle 03, sample 3.jpg”
4	1	2	3	“FT cycle 04, sample 1.jpg”	“FT cycle 04, sample 2.jpg”	“FT cycle 04, sample 3.jpg”
5	1	2	3	“FT cycle 05, sample 1.jpg”	“FT cycle 05, sample 2.jpg”	“FT cycle 05, sample 3.jpg”
6	1	2	3	“FT cycle 06, sample 1.jpg”	“FT cycle 06, sample 2.jpg”	“FT cycle 06, sample 3.jpg”
7	1	2	3	“FT cycle 07, sample 1.jpg”	“FT cycle 07, sample 2.jpg”	“FT cycle 07, sample 3.jpg”
8	1	2	3	“FT cycle 08, sample 1.jpg”	“FT cycle 08, sample 2.jpg”	“FT cycle 08, sample 3.jpg”
9	1	2	3	“FT cycle 09, sample 1.jpg”	“FT cycle 09, sample 2.jpg”	“FT cycle 09, sample 3.jpg”
10	1	2	3	“FT cycle 10, sample 1.jpg”	“FT cycle 10, sample 2.jpg”	“FT cycle 10, sample 3.jpg”

### 2.3 Data from modified version of the rolling bottle test

The data derived from the modified version of the Rolling Bottle Test (RBT) are reported in the folder “Data from modified version of RBT” which contains two files, namely “Weight of RBT specimens.xlsx” and the folder “RBT sample pictures”. The spreadsheet file “Weight of RBT specimens.xlsx” contains the weight of all the 588 RBT samples at three main stages: dried before the application of the additive, dried after the application of the additive and dried after testing considering 14 rotation time intervals (1 h, 2 h, 3 h, 4 h, 5 h, 6 h, 7 h, 8 h, 10 h, 12 h, 14h, 16 h, 20 h and 24 h, reported in columns B, T, AG, AY). Three replicate specimens are tested for each rotation time interval and they are sequentially named from “1” to “42”. The abbreviations of the tested stabilization technologies are reported in line 2. Based on these data, it is possible to assess the mass loss deriving from the modified version of the rolling bottle tests *ML_RBT_* (parameter defined in Section 2) as listed in Table 5.

**Table 5:** Experimental data of ML_RBT_ for each different binder technology.

	ML_RBT_ (%) for different rotation time intervals													
	1h	2h	3h	4h	5h	6h	7h	8h	10h	12h	14h	16h	20h	24h
UGM	0.41	0.78	0.88	1.04	1.33	1.49	1.70	1.94	1.98	2.06	2.31	2.40	2.85	3.03
CEM	2.82	3.09	3.30	3.52	3.60	3.69	3.70	3.74	3.94	4.21	4.32	4.42	4.81	5.11
BIT	0.01	0.08	0.10	0.24	0.29	0.31	0.46	0.59	0.74	0.85	0.97	1.19	1.51	1.75
SAL-A	2.39	2.67	2.94	3.32	3.84	4.04	4.37	4.77	4.86	4.89	5.03	5.12	5.65	6.32
SAL-B	2.74	3.12	3.27	3.48	3.57	3.71	3.88	4.09	4.34	4.43	4.66	4.72	4.87	4.90
BEN	1.16	1.54	1.57	1.62	2.08	2.16	2.24	2.31	2.70	2.89	2.95	3.23	3.46	4.11
LIG	2.38	2.66	2.87	3.04	3.18	3.47	3.53	3.92	4.08	4.29	4.54	4.60	4.90	5.15
SUG	2.30	2.52	2.82	3.11	3.14	3.42	3.63	3.71	3.91	4.01	4.43	4.75	5.07	5.62
RES	2.29	2.32	2.57	2.80	2.91	3.30	3.43	3.76	4.06	4.16	4.20	4.46	4.84	5.08
POL	0.21	0.24	0.27	0.29	0.25	0.29	0.30	0.31	0.32	0.35	0.39	0.41	0.55	0.62
ACR	0.53	0.58	0.71	0.86	0.94	0.99	1.23	1.34	1.75	1.90	1.98	2.37	2.66	2.92
STB	1.42	1.43	1.76	1.94	2.39	2.48	2.61	2.75	3.11	3.46	3.84	4.12	4.41	5.04
ACE-A	0.18	0.22	0.40	0.52	0.52	0.69	0.71	0.74	0.88	1.22	1.46	1.50	1.57	1.79
ACE-B	0.15	0.35	0.54	0.78	0.91	1.04	1.20	1.42	1.69	2.09	2.13	2.77	2.95	3.11

Furthermore, the folder “RBT sample pictures” contains 28 subfolders that display the images in .jpg format of each specimen before and after RBT. Consistently with the number of tested samples, each subfolder contains 42 images according to the designation reported in Table 6. In this way, it is also possible to visually assess the integrity with stripping loss for each stabilization technology and for each tested interval.

**Table 6:** Designation of RBT samples and corresponding images for each tested time interval.

Tested time	Specimen name			Name of corresponding image		
1 h	1	2	3	“m1.jpg”	“m2.jpg”	“m3.jpg”
2 h	4	5	6	“m4.jpg”	“m5.jpg”	“m6.jpg”
3 h	7	8	9	“m7.jpg”	“m8.jpg”	“m9.jpg”
4 h	10	11	12	“m10.jpg”	“m11.jpg”	“m12.jpg”
5 h	13	14	15	“m13.jpg”	“m14.jpg”	“m15.jpg”
6 h	16	17	18	“m16.jpg”	“m17.jpg”	“m18.jpg”
7 h	19	20	21	“m19.jpg”	“m20.jpg”	“m21.jpg”
8 h	22	23	24	“m22.jpg”	“m23.jpg”	“m24.jpg”
10 h	25	26	27	“m25.jpg”	“m26.jpg”	“m27.jpg”
12 h	28	29	30	“m28.jpg”	“m29.jpg”	“m30.jpg”
14 h	31	32	33	“m31.jpg”	“m32.jpg”	“m33.jpg”
16 h	34	35	36	“m34.jpg”	“m35.jpg”	“m36.jpg”
20 h	37	38	39	“m37.jpg”	“m38.jpg”	“m39.jpg”
24 h	40	41	42	“m40.jpg”	“m41.jpg”	“m42.jpg”

## 3 Experimental Design, Materials, and Methods

The tested rock aggregate material derives from Vassfjell, Heimdal, Norway. The thirteen binder technologies are obtained from industrial producers and the categories these additives belong to are representative of all the existing commercial products suitable to stabilize coarse aggregates [2], [3], [4], [5], [6], [7]. The testing campaign was performed in the laboratories of the Department of Civil and Environmental Engineering (Norwegian University of Science and Technology, Trondheim, Norway) to characterize the mechanical properties of unstabilized and stabilized rock aggregates.

As for road pavement engineering, the investigation and comparison of several stabilization technologies for construction aggregates is a very relevant topic to create more sustainable transportation infrastructures [22], [23], [24]. The major part of the roads worldwide comprises low-volume unpaved roads directly exposed to trafficking actions [25], [26], [27] suffering from poor maintenance and displaying several types of premature damage [28], [29], [30]. Considering the very scant amount of independent published literature regarding the comparison of different road stabilizers and the high relevance of the topic, the target audience is broad in nature. It encompasses transport agencies, contractors, industry and university researchers as well as companies manufacturing and supplying stabilization technologies [31]. In this regard, it may be worth mentioning that there is a myriad of proprietary road stabilizers available on the market globally, whose disclosed information is often subjective and rests on the laurels of the vendor's claims [32].

RLTTs were performed according to the Multi-Stage Low Stress Level (MS LSL) procedure; a RLTT is composed of thirty loading sequences as defined in the standard [33]. The analysed data can lead to the evaluation and comparison between all the binder treatments in terms of resilient modulus and resistance against permanent deformation. The particle size distribution used for the tests varied from 0 mm to 32 mm and thus corresponded to a typical road base layer; different quantities of binder ranging from 1% to 4% were mixed with 12 000 g aggregates for each RLTT sample [2,3]. The stress path applied is a combination of triaxial stress *σ_t_* and deviatoric stress *σ_d_* as reported in Table 7; each loading step applies 10 000 loading repetitions. A sequence is interrupted after the completion of the six loading steps or if the axial permanent deformation measured by the axial LVDT reaches 0.5%. Given a constant value of *σ_t_* and a variation in the dynamic deviatoric stress *Δσ_d,dyn_*, the resilient modulus *M_R_* is determined as

(1) $$M_{R}=\frac{\Delta \sigma_{d,dyn}}{\varepsilon_{a,el}},$$

where *ε_a,el_* is the mean axial resilient strain measured by the three axial LVDTs.

**Table 7:** Stress path for the Multi-Stage Low Stress Level (MSL SL) RLTT (data in kPa).

	Sequence 1		Sequence 2		Sequence 3		Sequence 4		Sequence 5	
	*σ_t_*	*σ_d_*	*σ_t_*	*σ_d_*	*σ_t_*	*σ_d_*	*σ_t_*	*σ_d_*	*σ_t_*	*σ_d_*
Step 1	20	20	45	60	70	80	100	100	150	100
Step 2	20	40	45	90	70	120	100	150	150	200
Step 3	20	60	45	120	70	160	100	200	150	300
Step 4	20	80	45	150	70	200	100	250	150	400
Step 5	20	100	45	180	70	240	100	300	150	500
Step 6	20	120	45	210	70	280	100	350	150	600

Marshall specimens containing the different binder technologies were created using the mould and laboratory procedure traditionally adopted for asphalt mixtures, with 50 compaction blows per side [34]. Each sample comprised 850 g aggregates with uniform coarse gradation ranging from 4 mm to 8 mm. The Marshall specimens were exposed to 10 FT cycles and the weight loss was measured after every cycle. Defining the dried mass of the sample recorded initially (*M_1_*) and after (*M_2_*) the selected amount of FT repetitions, the mass loss *ML_MRS_* is assessed as

(2) $$ML_{MRS}=\frac{M_{1}-M_{2}}{M_{1}},$$

and can be expressed as a percentage.

The procedures adopted to perform each FT repetition were: specimen submersion in water (23°C, 5 minutes), retrieval and release of water excess (23°C, 5 minutes), freezing (-15°C, 24 h) and thawing (23°C, 24 h for RLTT samples and 40°C, 48 h for Marshall samples) [2,3].

The RBT is originally a standardized procedure to evaluate the degree of adhesion between aggregate and bituminous binder covering the aggregate after the application of rotating and stirring actions [35]. The assessment is performed visually and therefore this gives room to possible unprecise results. As an improvement towards unequivocal interpretation, the testing campaign performed a modified version of RBT in the sense that the dried weight of a specimen covered by the additive was recorded before (*M_3_*) and after (*M_4_*) the testing while preserving the same rotating and stirring actions defined by the standard. In addition, the adjective “modified” also applies since several additive types were considered (and not only bituminous binder) and that 14 time intervals, namely 1 h, 2 h, 3 h, 4 h, 5 h, 6 h, 7 h, 8 h, 10 h, 12 h, 14h, 16 h, 20 h and 24 h, were evaluated (the code specifies to run the test only referring to 6 h and 24 h). The aggregates had size comprised between 8 mm and 11 mm; each RBT specimen was fabricated by blending 150 g of aggregates with 3% by mass of binder [2,3]. The mass loss *ML_RBT_* is assessed as

(3) $$ML_{RBT}=\frac{M_{3}-M_{4}}{M_{3}},$$

and can be expressed as a percentage.

## Ethics Statement

Ethical guidelines have been complied with during the collection of laboratory data. This work did not involve human subjects, animal experiments or data collected from social media platforms.

## CRediT authorship contribution statement

**Diego Maria Barbieri:** Conceptualization, Methodology, Software, Validation, Formal analysis, Investigation, Resources, Data curation, Writing – original draft, Visualization, Project administration. **Baowen Lou:** Conceptualization, Methodology, Software, Validation, Formal analysis, Investigation, Resources, Data curation, Writing – original draft, Visualization. **Robert Jason Dyke:** Conceptualization, Methodology, Formal analysis, Investigation, Resources, Data curation, Writing – review & editing. **Hao Chen:** Investigation, Resources, Writing – review & editing, Visualization. **Umesh Chandra Sahoo:** Visualization, Supervision. **Jeb S Tingle:** Visualization, Supervision. **Inge Hoff:** Conceptualization, Methodology, Writing – review & editing, Visualization, Supervision, Project administration, Funding acquisition.

## Declaration of Competing Interest

The authors declare that they have no known competing financial interests or personal relationships that could have appeared to influence the work reported in this paper.

## Data Availability

Mechanical properties of coarse aggregates stabilized with traditional and nontraditional additives: stiffness, deformation, resistance to freezing and stripping (Original data) (Mendeley Data).
